# Preliminary Establishment of a Method and System for Detecting Neural Tumor Boundaries Based on Optical Coherence Tomography

**DOI:** 10.1002/mco2.70498

**Published:** 2025-11-28

**Authors:** Jiuhong Li, Jinwei Li, Gonggong Lu, Feilong Yang, Jing Li, Xin Qi, Rui Zhang, Xiang Li, Jiachen Sun, Haibo Rao, Xuhui Hui, Si Zhang

**Affiliations:** ^1^ Department of Neurosurgery West China Hospital of Sichuan University Chengdu Sichuan China; ^2^ Department of Cardiovascular Surgery West China Hospital of Sichuan University Chengdu Sichuan China; ^3^ Department of Neurosurgery The Affiliated Santai Hospital of North Sichuan Medical College Mianyang Sichuan China; ^4^ Chengdu Incrpeak Optoelectronics Technology Co., Ltd. Central bldg. Optoelectric Industrial Park Chengdu Sichuan China; ^5^ Laboratory of Neurosurgical Institute West China Hospital of Sichuan University Chengdu Sichuan China; ^6^ School of Optoelectronic Science and Engineering of UESTC University of Electronic Science and Technology of China Chengdu Sichuan China

**Keywords:** intraoperative real‐time detection, live animal models, neural tumor boundary, optical coherence tomography, visualized reconstruction

## Abstract

Surgical intervention is vital for treating neural system tumors, with precise intraoperative determination of tumor boundaries crucial for safe and effective surgery. Optical coherence tomography (OCT), offering noninvasive, real‐time imaging, presents a promising solution. This study developed a swept‐source OCT system using a 1310 nm wavelength laser, enhanced by microelectromechanical systems technology for improved scanning accuracy. Neural tumor specimens and peritumoral tissues were analyzed, alongside evaluations in animal models, including rats and mice with gliomas, schwannomas, and meningiomas, to assess the system's real‐time surgical application. Results revealed significantly lower light attenuation in human glioma samples than in peritumoral tissues (*p* = 0.019), with an receiver operating characteristic curve area under the curve of 0.846. Gliomas exhibited higher pixel values and gentler trend line slopes (*p* < 0.001). Animal models showed the OCT system was capable of detecting nerves and their epineurium located deep to schwannomas and meningiomas (at depths <3 mm), which appeared as thin, tubular, or crescent‐shaped images with higher density compared with the surrounding tissue. These findings highlight the OCT system's ability to differentiate tumor from nontumoral tissues, demonstrating its potential as a handheld tool for precise boundary detection in neurosurgery. This advancement represents a promising step toward improving the accuracy of tumor resection.

## Graphical abstract

This diagram illustrates the stepwise workflow involved in the development and application of the intraoperative swept‐source OCT system for brain tumor detection. The process begins with the creation of the OCT system, followed by data collection on light attenuation values from brain tumors and their boundaries. The final step involves real‐time intraoperative visualization and testing of the system's effectiveness in detecting tumor boundaries in live animal models. The layout of Figure 1 was created with BioRender.

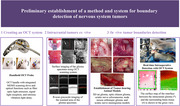

## 1 Introduction

The surgical treatment of intracranial tumors has entered the era of precision medicine, requiring neurosurgeons to achieve maximal tumor resection while preserving normal brain tissue to reduce tumor recurrence rates and ensure optimal postoperative neurological function [1]. Accurate intraoperative identification of tumor tissue and its boundaries is essential to achieving this goal. However, many intracranial tumors, particularly malignant ones, exhibit infiltrative growth patterns, making it difficult to distinguish clear boundaries from surrounding neural tissues and posing significant challenges to complete tumor resection [2]. Taking gliomas as an example, extended resection in nonfunctional areas is widely used in clinical practice to achieve maximal tumor removal whenever possible [3, 4]. However, when the tumor is located in critical functional areas such as the brainstem, basal ganglia, motor cortex, or language regions, complete resection cannot be achieved through extended surgery [5]. For brainstem gliomas, the incidence of postoperative complications ranges from 18 to 71%, including severe conditions like hemiplegia, swallowing dysfunction, and respiratory failure. The probability of permanent neurological deficits after surgery can be as high as 29% [6, 7, 8]. Therefore, maximizing the extent of tumor resection while preserving essential functional areas and cranial nerves is crucial for patients’ progression‐free survival and postoperative quality of life. Effective intraoperative identification of the tumor and its boundaries is vital to achieving these outcomes.

Currently, the determination of intraoperative brain tumor boundaries primarily relies on visual identification under a microscope or neuroendoscope [9]. However, this approach is highly subjective and lacks accuracy. Even for experienced neurosurgeons, it is challenging to delineate the boundaries of tumors with unclear margins during surgery. The gold standard for detecting intracranial tumors and their boundaries is intraoperative frozen pathology [10]. However, this technique has several limitations, including discontinuity, long waiting times, and the fact that it can only assess limited, point‐specific tissue samples. Additionally, it is unsuitable for lesions in critical functional areas, such as the brainstem or central region, as tissue sampling may cause bleeding, severe neurological deficits, or even death [11]. Another widely used method for detecting intracranial tumors and boundaries is intraoperative neurophysiological monitoring [12]. However, this method cannot reliably distinguish small residual tumor tissues on the surface of functional areas and is prone to false positives and false negatives [13]. In summary, there is currently a lack of real‐time, high‐resolution, and high‐precision imaging technology capable of continuous monitoring to assist surgeons in accurately determining the boundaries between tumors and normal brain tissue during surgery.

Optical coherence tomography (OCT) is a noninvasive, low‐cost imaging technology that does not require contrast agents and enables real‐time three‐dimensional (3D) imaging of tissue structures [14, 15]. In recent years, researchers have begun applying OCT to detect neural tissue specimens and assist in distinguishing between neural tumors and normal brain tissue [16, 17, 18, 19]. In 2015, Kut et al. used quantitative OCT to measure fresh human glioma and peritumoral nontumoral brain tissue specimens. They determined that a light attenuation value of 5.5 mm^−^^1^ could serve as a diagnostic threshold for distinguishing gliomas from peritumoral nontumoral tissue, with a sensitivity of 92–100% and a specificity of 80–100% [14]. In 2019, Juanrez‐Chambi et al. collected ex vivo samples from 21 patients and employed the Canny edge detection algorithm to overcome the fixed detection distance requirement for specimens, thus achieving boundary recognition. Using a machine learning algorithm, they found that the OCT system had a sensitivity of approximately 90% and a specificity of 80% in differentiating glioma‐infiltrated brain tissue from noninfiltrated tissue [20]. However, the algorithm failed to account for the variable distance between the OCT probe and the specimen surface, which could affect important parameters such as OCT grayscale and attenuation values [21]. Surgeons often need to remove a volume of at least 5 × 2 × 2.5 mm^3^ to ensure thorough tumor resection, but if this region is located in critical functional areas such as the brainstem, motor cortex, or language centers, removing such a large volume may result in severe neurological deficits. In summary, studies on the application of OCT for detecting neural tissues and identifying neural tumor boundaries are limited and have certain shortcomings. No systematic research has been conducted on this topic.

**FIGURE 1:**
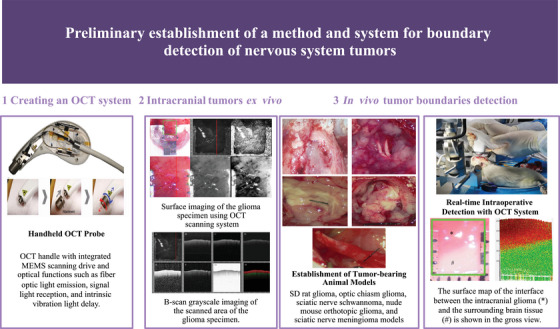
Schematic representation of the technical workflow for the intraoperative OCT system. This diagram illustrates the stepwise workflow involved in the development and application of the intraoperative swept‐source OCT system for brain tumor detection. The process begins with the creation of the OCT system, followed by data collection on light attenuation values from brain tumors and their boundaries. The final step involves real‐time intraoperative visualization and testing of the system's effectiveness in detecting tumor boundaries in live animal models. The layout of this figure was created with BioRender.

This study aims to develop a hardware system based on OCT technology for real‐time intraoperative detection of neural system tumors and boundaries and to optimize a portable handheld OCT probe using microelectromechanical systems (MEMS) technology (Figure 1). A custom software system based on Visual C++ 6.0 will be developed to quantify and visualize OCT grayscale images. By collecting fresh, pathologically confirmed ex vivo neural system tumor tissues and peritumoral nontumoral brain tissues, we will establish an OCT grayscale image database for various neural system tumor and nontumoral tissues. Finally, multiple tumor‐bearing animal models (including rat glioma models, optic chiasm glioma models, sciatic nerve schwannoma models, as well as nude mouse glioma and sciatic nerve meningioma models) will be constructed to validate the effectiveness and practicality of the OCT system in detecting tumor boundaries in vivo.

## 2 Results

### 2.1 Establishment of an Integrated Hardware–Software System for Intraoperative Brain Tumor Boundary Detection Based on OCT

A 1310 nm swept‐source laser (HSL‐20) based handheld OCT probe scanning system was established for intraoperative neural tumor boundary detection. The OCT scan lens enabled a single B‐scan to probe an 11 mm depth and 5 mm width perpendicular to the probe. With a spectral bandwidth Δ*λ* = 100 nm, the 1310 nm system achieved a theoretical axial resolution of 7.6 µm in air. Each A‐line was acquired in 40 ms, yielding ≥ 2000 spatially resolved spectral back‐scattering data points. The laser coherence frequency was stabilized at 30 kHz, with laser power ≤ 40 mW (peak) and ≤15 mW (average), and a custom MEMS mirror raster‐scanned the *X*–*Y* plane in 1.2–2.4 s per volume, enabling rapid tissue imaging (Figure 1).

**FIGURE 2:**
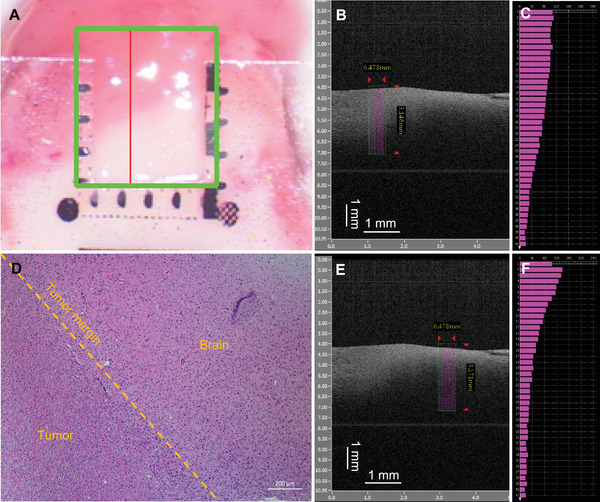
Self‐built OCT software reveals a representative grayscale B‐scan and gray‐value sampling profile from a pathologically confirmed glioma specimen containing peritumoral nontumoral brain tissue. The gross specimen (A) displays a faint‐pink tumor superiorly and a white nontumoral zone inferiorly. The red line on the specimen surface indicates the scanning plane, corresponding to the grayscale B‐scan images (B and E). Purple sampling lines, covering identical width and depth, were placed on the B‐scans (B and E) and plotted in (C) and (F). The tumor region (C) exhibits a lower mean gray‐value peak than the peritumoral tissue (110 vs. 140) and a slower decline in gray‐value with depth. HE staining (D) confirms the tumor and nontumor regions; the tumor margin corresponds to the red–white junction on the gross specimen (A).

Building upon analyses of swept‐source OCT data processing and image reconstruction, an automated acquisition and reconstruction software system was successfully implemented in the Visual C++ 6.0 environment (Figure 1). A region‐of‐interest (ROI) sampling line mode for grayscale images was designed to acquire and quantitatively display the mean gray‐value profile as a function of imaging depth (Figure 2).

### 2.2 Measurement of OCT Grayscale Values and Data Analysis for Different Tumors and Tissues

A total of 20 intracranial neural system tumor patients were included in the study, from whom tumor tissue and peritumoral tissue were collected intraoperatively. A total of 103 fresh ex vivo specimens were measured, with multiple specimens collected and scanned from some patients. Among the 20 patients, 14 had gliomas. Of these, five had WHO grade 2, three had grade 3, and six had grade 4 gliomas; additionally, two patients had metastatic tumor and one had lymphoma. There were also three specimens of normal cerebellar tissue from three patients (Table S1).

**FIGURE 3:**
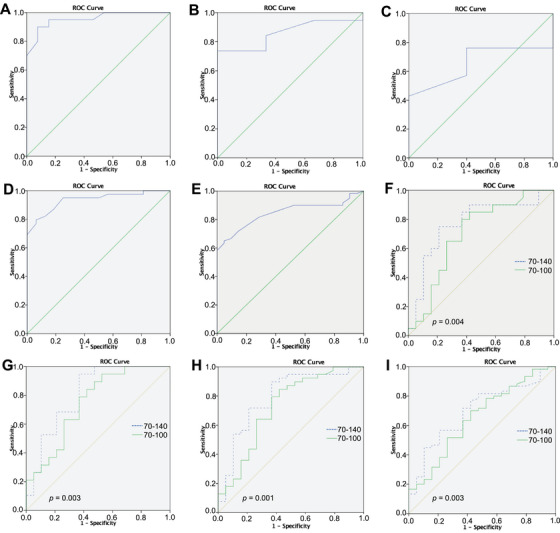
ROC curves of optical attenuation coefficients and pixel‐based grid analyses for supratentorial gliomas versus peritumoral nontumoral brain tissue. (A) WHO grade 4 glioma versus peritumoral tissue. (B) WHO grade 3 glioma versus peritumoral tissue. (C) WHO grade 2 glioma versus peritumoral tissue. (D) Combined WHO grades 3 and 4 gliomas versus peritumoral tissue. (E) Combined WHO grades 2, 3, and 4 gliomas versus peritumoral tissue. (F) WHO grade 4 glioma versus peritumoral tissue (*p* = 0.004). (G) WHO grade 3 glioma versus peritumoral tissue (*p* = 0.003). (H) Combined WHO grades 3 and 4 gliomas versus peritumoral tissue (*p* = 0.001). (I) Combined WHO grades 2, 3, and 4 gliomas versus peritumoral tissue (*p* = 0.003). In panels (F)–(I), ROC curves obtained with a 70–140 attenuation threshold (blue dashed line) exhibit larger areas under the curve (AUC) than those generated with a 70–110 threshold (green solid line).

The distance between the OCT probe and the specimen was 2.22 ± 0.89 mm (range, 0.4–4.6 mm) in the supratentorial gliomas tissues (*n* = 60) and 2.08 ± 0.86 mm (range, 0.7–4.0 mm) in the peritumoral normal brain tissues (*n* = 21); the difference was not statistically significant (*p* > 0.05). For WHO grade 2, 3, and 4 gliomas, as well as high‐grade and low‐grade supratentorial gliomas, the average light attenuation values were significantly lower compared with those of peritumoral nontumoral brain tissues (*p* < 0.05) (Table 1). The receiver operating characteristic (ROC) curves of optical attenuation coefficients were depicted in Figure 3A–E. The ROC curve analysis results for the light attenuation values of supratentorial gliomas and peritumoral normal brain tissues are summarized in Table S2. Additionally, the specimens were divided into two groups based on the distance between the OCT probe and the specimen, using 1.5 mm as the threshold for subgroup analysis. In the subgroup where the distance was less than or equal to 1.5 mm. The OCT grayscale A‐line and trendline were plotted and measured for the specimens. The statistical analysis results for the A‐line and trendline of supratentorial gliomas and peritumoral nontumoral brain tissues are presented in Table S3. The mean angle between the descending segment of the trend line and the vertical axis was significantly greater in gliomas (18.44 ± 7.27° overall, 18.10 ± 6.98° in the ≤1.5 mm subgroup, and 18.60 ± 7.50° in the >1.5 mm subgroup) than in peritumoral nontumoral brain tissue (12.24 ± 6.08°, 11.76 ± 4.04°, and 12.24 ± 6.08°, respectively; all *p* < 0.01).

**TABLE 1:** Statistical results of optical attenuation coefficients in ex vivo human brain specimens.

Tissue type	*n*	Optical attenuation coefficient (mean ± SD, mm^−^^1^)	*p* Value
Supratentorial WHO grade 4 glioma			
Tumor region	20	4.62 ± 0.98	<0.001
Peritumoral nontumoral tissue	13	6.49 ± 0.59	−
Supratentorial WHO grade 3 glioma			
Tumor region	19	4.50 ± 1.47	0.004
Peritumoral nontumoral tissue	3	6.03 ± 0.45	−
Supratentorial WHO grade 2 glioma			
Tumor region	21	5.28 ± 1.96	0.019
Peritumoral nontumoral tissue	5	6.38 ± 0.35	−
Supratentorial high‐grade glioma^a^			
Tumor region	39	4.56 ± 1.23	<0.001
Peritumoral nontumoral tissue	16	6.41 ± 0.58	−
Supratentorial high‐ and low‐grade glioma^b^			
Tumor region	60	4.81 ± 1.51	<0.001
Peritumoral nontumoral tissue	21	6.40 ± 0.53	−
Cerebellar glioma			
Diffuse astrocytoma of vermis	1	3.8	−
Normal cerebellar tissue	3	4.17 ± 0.44	−
Temporal lobe metastatic lung adenocarcinoma			
Tumor region	1	5.45	−
Peritumoral nontumoral tissue	2	4.20 ± 0.57	−
Cerebellar hemisphere metastatic lung adenocarcinoma			
Tumor region	9	5.14 ± 0.68	0.004
Peritumoral nontumoral tissue	3	6.67 ± 0.21	−
Parietal lobe lymphoma			
Tumor region	1	4.5	−
Peritumoral nontumoral tissue	2	2.85 ± 0.49	−

*Abbreviations*: SD, standard deviation; WHO, World Health Organization.

^a^ Supratentorial high‐grade glioma: combined data of WHO grade 3 and grade 4 gliomas.

^b^ Supratentorial high‐ and low‐grade glioma: combined data of WHO grade 2, 3, and 4 gliomas.

The ROC curves of the mean pixel count within 55 × 100‐pixel region were depicted in Figure 3F–I. Within the 55 × 100‐pixel region, the mean pixel count falling into the 70–140 grayscale interval was significantly higher in gliomas (1409.47 ± 478.08) than in peritumoral nontumoral tissue (1099.11 ± 333.10; *p* = 0.003). ROC curve metrics derived from these pixel‐based grid analyses for supratentorial gliomas versus peritumoral nontumoral brain tissue are summarized in Table S4.

### 2.3 3D Visualization Based on Actual OCT Scanning Regions

**FIGURE 4:**
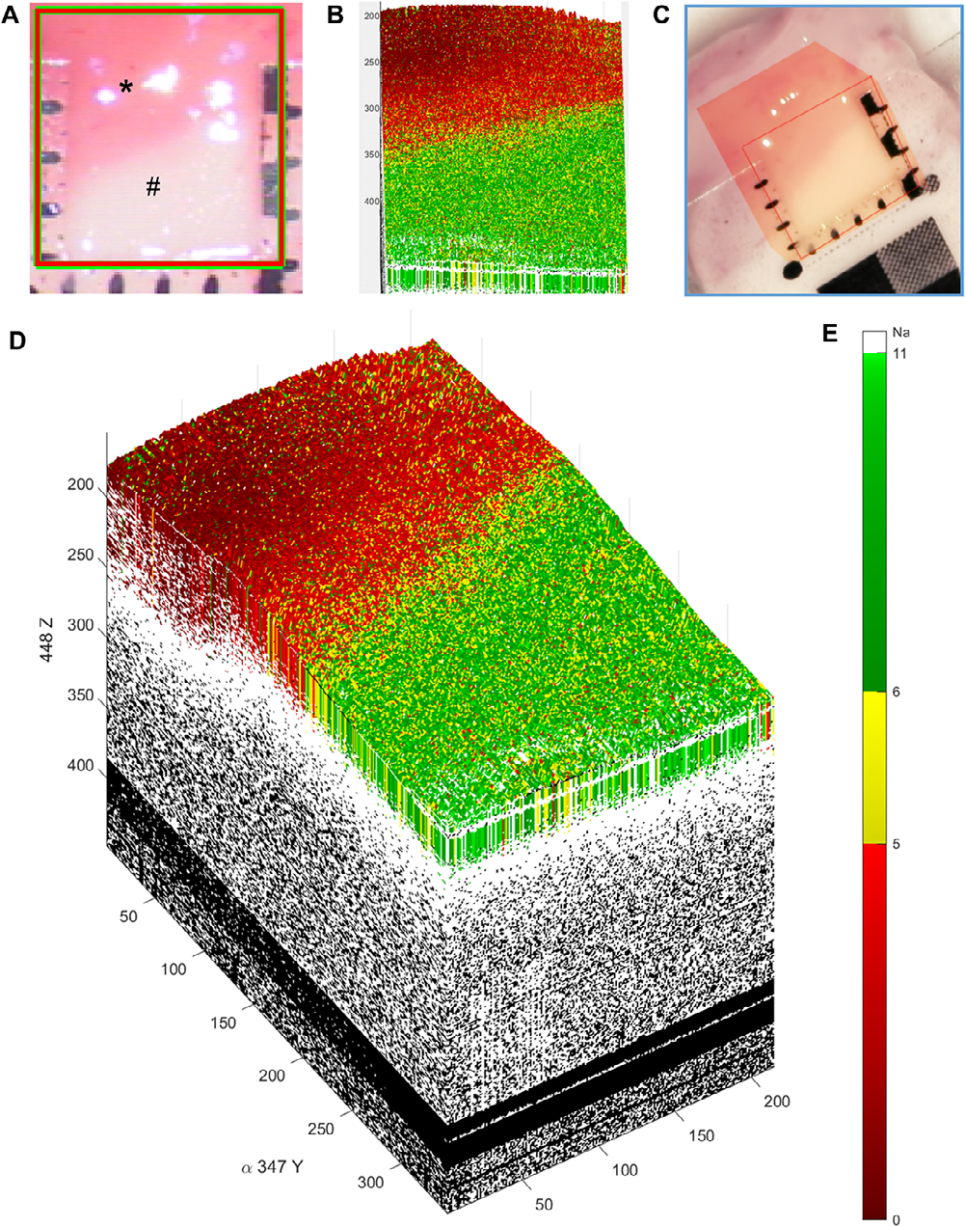
Surface and 3D visualization reconstruction of OCT grayscale images from glioma boundary specimens. (A) A macroscopic surface view of the boundary region between the supratentorial glioma (*) and peritumoral brain tissue (#), confirmed by pathological examination. (B) A reconstructed surface map generated using MATLAB software, where red regions represent tumor tissue, green regions indicate peritumoral brain tissue, and yellow regions correspond to the tumor–brain tissue boundary. (C and D) Three‐dimensional grayscale image reconstruction of the analyzed region. The software automatically assigns attenuation values of 0–5 as red (indicating a high probability of tumor tissue), 6–11 as green (suggesting peritumoral brain tissue), and 5–6 as yellow (representing the tumor–brain tissue boundary). This reconstruction approach provides detailed and intuitive visualization of glioma boundaries.

Using MATLAB software, a total of 215 grayscale images were input from the MEMS system after performing 10 back‐and‐forth scans of a specimen (5 × 5 × 10.95 mm^3^). The grayscale matrix data corresponding to each OCT image were identified and then stitched together to form a 3D grayscale block. The surface and sides of the 3D block were color coded to achieve 3D visualization and reconstruction. The reconstruction results are shown in Figure 4. The entire 3D reconstruction process took only 3.4 s, including data preprocessing, image saving, data saving, data reading, and image rendering.

### 2.4 Application of the OCT System in Tumor Boundary Detection in Animal Models

**FIGURE 5:**
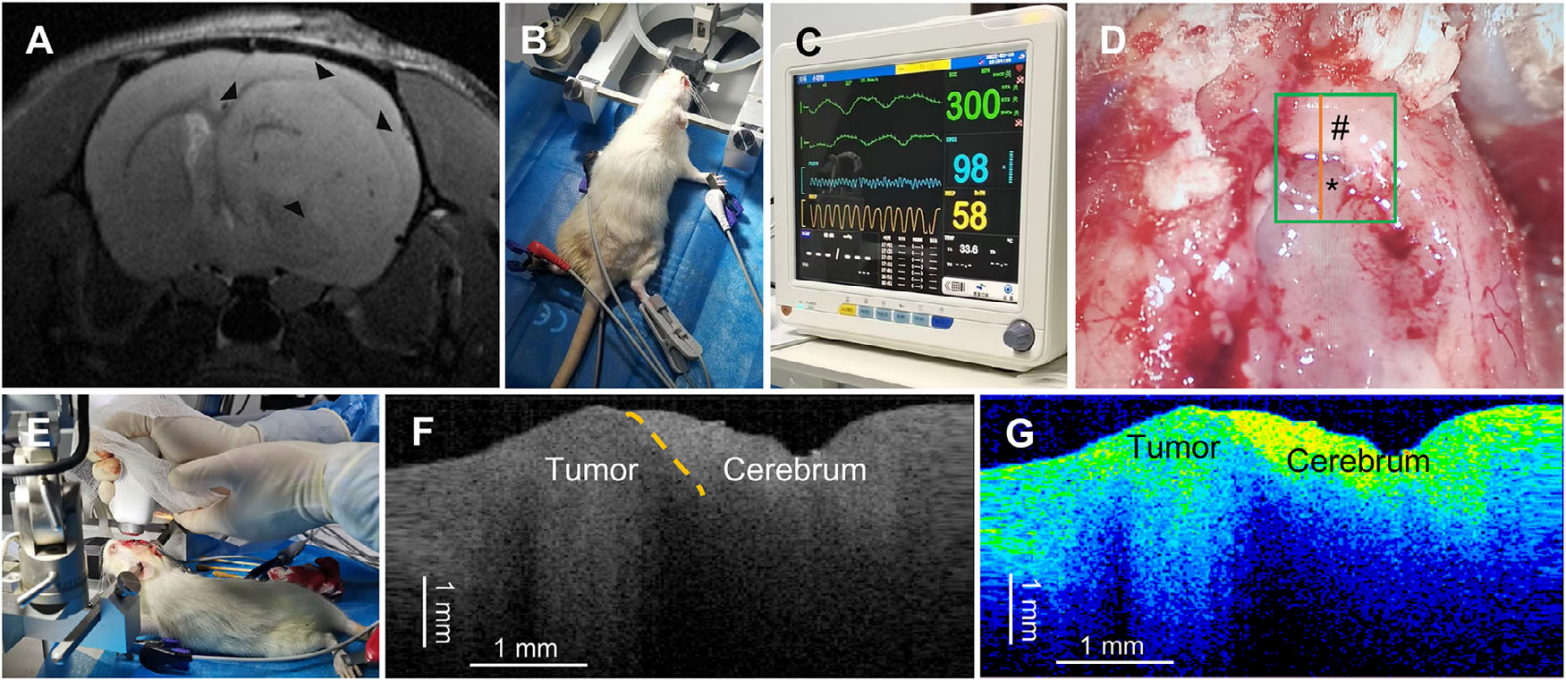
Application of the OCT system for intraoperative boundary detection of supratentorial gliomas in an SD rat model. (A) Preoperative T2‐weighted magnetic resonance imaging (MRI) of the SD rat supratentorial C6 glioma model shows a right frontal lobe lesion (triangular arrow). (B) The animal was securely positioned following induction of anesthesia. (C) Basic vital signs, including ECG waveform, heart rate, oxygen saturation, respiratory rate, and body temperature, were monitored using a cardiac monitor. (D) After craniotomy, the bone window was removed, and brain tissue covering the tumor was resected to expose the tumor area. The region of interest (green box) was then subjected to real‐time, contact‐free OCT detection. (E) The OCT system was used for imaging the exposed area. (F) A B‐scan grayscale image of the region showed the boundary between the tumor and peritumoral normal brain tissue (indicated by the orange dashed line). The low‐density area on the left of the dashed line corresponds to the tumor, while the high‐density area on the right corresponds to the peritumoral normal brain tissue. (G) A two‐dimensional color map reconstructed using ImageJ software highlights the tumor region with low‐intensity color (blue and green) and the peritumoral normal brain tissue with higher‐intensity color (red and yellow). Note, red, orange, and yellow denote high gray‐scale values; green indicates an intermediate level; blue and black correspond to low gray‐scale values. Copyright permission from our previously published work Ref. [24].

The OCT system was applied to detect the boundaries of supratentorial gliomas, optic chiasm gliomas, brainstem gliomas, and cerebellar gliomas. The working distance between the OCT probe and the specimen was consistently around 1.5 mm across all regions. Based on the imaging differences between the tumor and peritumoral normal tissues (brain parenchyma or optic nerve), the OCT system can assist in the real‐time intraoperative differentiation of tumor tissue from normal brain tissue. Preoperative head MRI T2‐weighted images (Figure 5A) showed a mass in the right frontal lobe. The anesthetized rat was immobilized, and its vital signs were monitored (Figure 5B, C). After detection, the tissue type of the scanned area was confirmed by HE staining (Figure 5D, E). According to the OCT grayscale images, C6 gliomas exhibited low or slightly lower density shadows, while the peritumoral normal brain tissue showed high or slightly higher density shadows (Figure 5F). The two‐dimensional (2D) color images reconstructed using ImageJ indicated that C6 gliomas had predominantly lower color scale characteristics (green and blue), while peritumoral normal brain tissue exhibited higher color scale characteristics (red and yellow) (Figure 5G).

**FIGURE 6:**
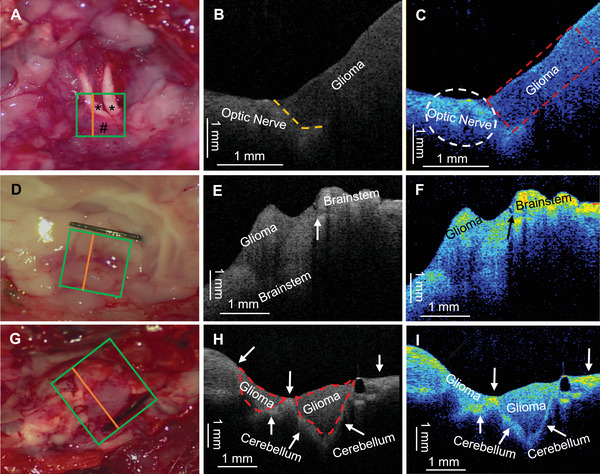
Intraoperative OCT mapping of infiltrative glioma margins in rat models in vivo: optic chiasm (A–C), brainstem (D–F), and cerebellum (G–I). (A) The bilateral optic nerves (*) and the glioma (#) originating from the optic nerve were exposed. (B) One B‐scan grayscale image (orange segment in A) reveals a boundary (orange dashed line in A) between the low‐density area in the upper‐right region (glioma) and the high‐density area in the lower‐left region (optic nerve), clearly delineating the glioma and optic nerve boundary. (C) The reconstructed two‐dimensional color map highlights the tumor region (red box) with predominantly low‐intensity colors (black and blue), while the exposed optic nerve and the optic nerve covered by the glioma (white elliptical circle) exhibit predominantly high‐intensity colors (green and yellow). The contrast between these regions is particularly evident at the tumor–nerve boundary. (D) After gross total resection of the glioma, it is difficult to determine accurately whether there was residual tumor on the brainstem surface under the operative microscope. (E) One B‐scan grayscale image (orange segment in D) shows the tumor area as a low‐density region and the peritumoral brainstem tissue as a high‐density region, clearly identifying the tumor–brainstem interface (arrow). (F) The reconstructed two‐dimensional color map indicates that the brainstem surface adjacent to the tumor exhibits high‐intensity colors (red and yellow) compared with the tumor region. (G) After tumor resection of the cerebellar glioma, the OCT system was used to detect the tumor boundary. (H) One B‐scan grayscale image (orange segment in G) reveals a thin, high‐density layer (arrows) surrounding a low‐density region (two irregular red dashed areas), suggesting the tumor location. (I) The reconstructed two‐dimensional color map reveals cerebellar tissue (green and yellow high‐intensity colors) slightly deep to residual tumor, enabling precise prewarning of tumor margins and thus enhancing intraoperative resection safety and accuracy. Note, red, orange, and yellow denote high gray‐scale values; green indicates an intermediate level; blue and black correspond to low gray‐scale values.

After resection of most of the bilateral frontal lobes, both optic nerves and the glioma originating from the optic nerves are visible (Figure 6A). The OCT system scanned the ROI, and the surface grayscale image indicated that the optic nerves were located at the top, confirming accurate localization of the ROI (Figure 6B). The optic nerves also appeared as high‐density shadows in the OCT grayscale images. When the remaining tumor layer was thin (approximately 2 mm), the OCT system detected the normal brain tissue or optic nerve deep to the thin tumor layer, providing a warning about the presence of deep normal tissue and the optic nerves (Figure 6C). The visualized images also clearly delineated the boundary between the tumor and the optic nerve (Figure 6B, C).

Subsequently, the OCT system was evaluated for intraoperative boundary detection of brainstem gliomas in (Sprague Dawley) SD rats. Partial tumor resection was performed, debulcking and thinning the tumor tissue overlying the brainstem surface (Figure 6D). Subsequent OCT scanning showed that the tumor region appeared as a low‐density area, while the surrounding brainstem tissue was of higher density, suggesting that the high‐density area was the brainstem tissue adjacent to the tumor (Figure 6E). The reconstructed 2D color visualization revealed higher color scales attached to the surface of the brainstem compared with the tumor region. This visualization highlighted the boundary between the brainstem and the tumor (Figure 6F).

In an SD rat model with a C6 glioma in the cerebellum, after near total resection of the glioma, the attachment of the glioma was revealed (Figure 6G). The OCT system was used to detect the tumor boundary, and the grayscale image showed a thin, high‐density shadow surrounding a low‐density region, suggesting the tumor area (Figure 6H). The 2D color image displayed signs of high color scale enrichment on the surface of the peritumoral normal cerebellar tissue. In the deeper regions of the peritumoral normal cerebellar tissue, there was an area of higher color scale enrichment compared with the upper region adjacent to the tumor, where blue was more prevalent (Figure 6I).

**FIGURE 7:**
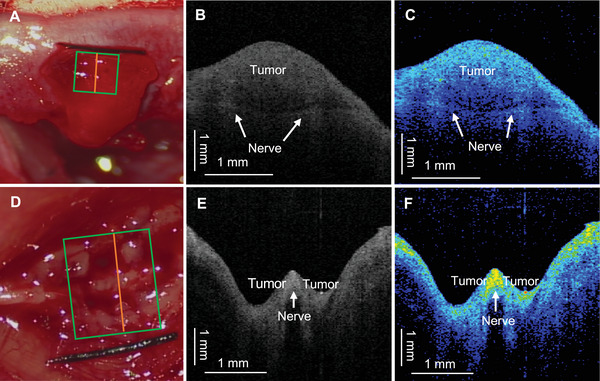
Intraoperative in vivo OCT delineation of adhesive tumor margins. (A–C) RT4 schwannoma in an SD rat; (D–F) sciatic‐nerve meningioma in a nude mouse. (A) After partial tumor resection, leaving a thin layer of tumor tissue (1–2 mm) on the nerve, the sciatic nerve was positioned to a superficial location for OCT scanning after severing the distal segment to ensure better detection depth. (B) One B‐scan grayscale image (orange segment in A) reveals a slightly higher‐density layered shadow (arrows) below the low‐density shadow, corresponding to the epineurium of the sciatic nerve as revealed in OCT imaging. (C) The two‐dimensional color map shows a blue layered signal shadow (arrows) matching the surface color scale. (D) The IOMM meningioma tumor covering the surface of the sciatic nerve was near totally resected, leaving only a thin layer of residual tumor. (E) One B‐scan grayscale image (orange segment in D) displays the sciatic nerve as a tubular high‐density shadow (arrow) with a strip‐like low‐density shadow beneath it. The surrounding tumor tissue appears as a low‐density shadow. (F) The two‐dimensional color map highlights a high‐intensity signal enriched on the surface of the sciatic nerve, while the surrounding areas predominantly show low‐intensity signals, delineating the boundary between the meningioma and the sciatic nerve. Note, red, orange, and yellow denote high gray‐scale values; green indicates an intermediate level; blue and black correspond to low gray‐scale values.

The application of the OCT system in boundary detection of the (RT4‐D6P2T cell) RT4 sciatic nerve tumor model is shown in Figure 7A–C. In the RT4 sciatic‐nerve tumor model of SD rats, a spherical tumor formed along the right sciatic nerve, with the nerve coursing immediately beneath the neoplastic mass. In this RT4 sciatic schwannoma model, due to the short‐term growth of the tumor, the sciatic nerve's epineurium remained mostly intact. This model closely resembles the positional and adhesive relationship between the tumor and the facial nerve in patients with vestibular schwannoma. The OCT system was used to detect live tissue specimens from the schwannoma animal model. After near total resection of the tumor, a thin layer of tumor tissue (1–2 mm) remained on the nerve. Given the depth of the detection, the distal sciatic nerve was transected and placed in a superficial position (Figure 7A). The grayscale image of the ROI showed a slightly higher‐density layered shadow beneath the low‐density tumor area (Figure 7B). This corresponds to the OCT imaging characteristics of the sciatic nerve's epineurium, with the higher‐density shadow indicating the sciatic nerve covered by the schwannoma, effectively demonstrating the boundary between the schwannoma and the sciatic nerve. The 2D color image displayed a higher layered signal shadow (blue), compared with surrounding tumor tissue (Figure 7C). Subsequently, the OCT system was used intraoperatively to detect live tissue in a sciatic nerve meningioma animal model in nude mice (Figure 7D–F). In a nude mouse bearing a right‐sided sciatic‐nerve intraosseous meningioma (IOMM), the tumor enveloping the nerve was debulked, leaving only a thin residual layer. The ROI was subsequently imaged using the OCT system (Figure 7D). The grayscale image (Figure 7E) displayed the sciatic nerve as a tubular high‐density shadow, with a strip‐like low‐density shadow underneath, and the surrounding tumor tissue as a low‐density shadow. The 2D color image showed pronounced high‐color‐scale signal concentrated within the sciatic nerve, whereas the surrounding meningioma exhibited predominantly lower color scales, sharply demarcating the nerve–tumor interface (Figure 7F).

### 2.5 Data Analysis of Boundary Detection in Live Tumor‐Bearing Animal Models Using the OCT System

The OCT system was used to detect a total of 71 pathologically confirmed tumor samples and 71 peritumoral normal tissue samples in vivo. The total pixel count within the 100–140 grayscale range was measured using the grid method in a 55 × 80 (width × height) grid area (Table S5). Compared with peritumoral normal brain tissue, the grayscale values were significantly lower for supratentorial C6 tumors, optic chiasm C6 tumors, brainstem C6 tumors, basal ganglia C6 tumors, and supratentorial U87 tumors (*p* < 0.05).

The average grayscale values were also calculated using the mean grayscale value method for a specific width and depth, with the statistical results presented in Table S6. The results showed that the grayscale values for supratentorial C6 tumors, brainstem C6 tumors, and supratentorial U87 tumors were statistically lower than those of peritumoral normal brain tissue (*p* < 0.05).

## 3 Discussion

In this study, we developed an OCT‐based system for real‐time detection of neural system tumors and their boundaries. Compared with existing intraoperative imaging techniques, the OCT system offers advantages such as noncontact operation, high resolution, and real‐time imaging, making it particularly suitable for neurosurgical applications [22, 23, 24]. Current intraoperative imaging methods, including diffusion tensor imaging, intraoperative MRI, fluorescence labeling, and intraoperative ultrasound, are widely used but have limitations in boundary detection accuracy, imaging resolution, and real‐time imaging capability [25, 26, 27, 28, 29]. Additionally, some methods, such as intraoperative MRI, are costly and require bulky equipment, which limits their accessibility in most domestic operating rooms. Through various animal tumor models established in this study, we validated the effectiveness and scientific robustness of the OCT system for boundary detection, particularly in models of brainstem gliomas and basal ganglia‐invading gliomas. These findings indicate that the OCT system can significantly support precision surgery for gliomas in critical functional areas, providing a solid foundation for further clinical application research based on OCT technology.

Among fluorescence staining techniques, 5‐aminolevulinic acid is the most commonly used. However, it has low specificity (53–96%) and sensitivity (75–95%) and relatively poor resolution. Furthermore, it is less effective in surgeries for low‐grade gliomas and is costly [30]. Intraoperative ultrasound, while useful, requires direct contact with brain tissue, which can exert pressure on the tissue and potentially cause damage during imaging [31]. The large size of ultrasound probes makes it difficult to apply to smaller lesions or deep‐seated tumors. Its resolution is at the millimeter level, making it difficult to detect small clusters of tumor cells, whose diameters may be at the micrometer level [32, 33, 34, 35, 36, 37, 38]. Additionally, ultrasound does not provide objective, quantifiable tissue data and is subject to significant operator bias during diagnosis. Intraoperative MRI offers noninvasive and precise imaging, but it has significant limitations. The long scanning times result in reduced spatial resolution, and it cannot provide real‐time imaging during surgery [38, 39, 40, 41]. Furthermore, certain patients, such as those with pacemakers or other magnetic metal implants, cannot undergo MRI, and the cost of MRI diagnostics is significantly higher than other imaging techniques [42]. The heavy and expensive equipment also makes it inaccessible for most domestic operating rooms, limiting its availability.

All human specimens examined in this study were derived from fresh ex vivo tissue. The cohort comprised a broad spectrum of intracranial malignancies—including gliomas, cerebellar metastases, and lymphomas—together with corresponding peritumoral, nontumoral brain tissues. Kut's study neither included peritumoral nontumoral brain tissue from patients with WHO grade 2 tumors nor specimens containing both neoplastic and nontumoral brain tissue [14]. Our study innovatively proposes two parameters, which were A‐line trend‐line and grid‐based method to analyze OCT grayscale images. Because the trend‐line derives its values from the averaged intensities of selected rows, it achieves greater stability than single A‐line analysis in identifying peak locations, descending slopes, and vertical angles, thereby compensating for the inherent instability of the conventional A‐line approach. The grid metric, introduced here for the first time, quantifies the sum of pixels whose grayscale values fall within a specific range (70–140 or 100–140) over a predefined region. This parameter reflects both the mean grayscale intensity and the distribution characteristics of the tissue. Its principal advantage lies in its suitability for delineating the transitional zone between tumor and surrounding normal brain. We have developed an OCT tissue‐visualization platform in MATLAB that ingests batches of grayscale OCT images, automatically extracts their gray‐level data, stitches multiple images into a 3D volume, and integrates attenuation calculation, comparative analysis, and visualization into one seamless workflow. High‐contrast, color‐graded maps (red → yellow → green) and an intuitive 3D reconstruction are generated within seconds. The resulting volumetric display preserves the orientation and viewing angles familiar to surgeons during an operation. Compared with the limited published reconstructed OCT systems [14, 20, 43, 44, 45, 46, 47], our 3D visualization suite offers three distinct advantages. (1) High‐resolution 3D reconstruction. By applying an interleaving algorithm to reorder the 215 grayscale images acquired during five bidirectional MEMS sweeps, we reduced the slice‐to‐slice interval from 0.187 to 0.0187 mm, which was higher than the 0.033 mm interslice spacing reported for the 3D reconstruction system developed by Kut et al. (2) The innovative gradient‐display modality in our 3D reconstruction system provides an intuitive intraoperative alert. As the rendered volume transitions toward vivid red, it signals that the resection plane is approaching the tumor margin and adjacent peritumoral brain tissue—prompting the surgeon to proceed gently and avoid injuring the normal parenchyma that lies deep to the tumor. (3) Our 3D reconstruction employs a curved‐surface mesh that faithfully follows the undulating topography of the specimen and is subsequently color‐mapped. This approach preserves surface concavities and convexities, yielding images that closely match the surgeon's intraoperative view, thereby facilitating precise localization of the interrogated region and any sub‐domains within it. Compared with the reconstruction of Juarez‐Chambi et al. [20], which flattens irregular surfaces into uniformly planar volumes, our method offers a clear advantage in topographic fidelity.

OCT‐microangiography can delineate tumor margins by mapping microvascular density, yet in highly vascularized tumors the peritumoral brain parenchyma itself often exhibits abnormally dense vasculature, which may lead to false‐positive boundary identification [48]. Optical coherence elastography (OCE) leverages the micrometer resolution and phase sensitivity of OCT to quantify the tissue's Young's modulus distribution during controlled deformation, thereby translating elastic contrast into stiffness maps for morphological segmentation. Nevertheless, this approach requires direct probe contact and compression, which may increase the risk of cerebral parenchymal injury and thus limit its translational value for intraoperative tumor‐border delineation in neurosurgery [49, 50]. Subsequently, Burhan et al. have combined OCE with an air‐pulse nozzle to discriminate tumor types; the technique still relies on applying a controlled mechanical load to the tissue so that its stiffness can be inferred. Whether this mechanical interaction can induce secondary damage—such as edema—in the peritumoral brain parenchyma remains to be validated in vivo [51].

In this study, we developed several animal models for tumor boundary detection using the OCT system, including brainstem glioma, optic nerve glioma, sciatic nerve schwannoma, and peripheral nerve meningioma models. To date, there have been no related reports in the literature using OCT to detect tumor boundaries in these models. We established two major categories of tumor‐bearing animal models. The first category includes infiltrative growth models, such as the rat brain glioma model, the rat optic chiasm glioma model, and the nude mouse human brain glioma model. The second category includes adhesive growth models, such as the rat peripheral nerve malignant schwannoma model (including sciatic nerve schwannoma models) and the nude mouse peripheral nerve malignant meningioma model. These models were designed to simulate clinical conditions involving human brain parenchymal gliomas, optic pathway gliomas, schwannomas, and meningiomas. They were used to evaluate the effectiveness and practicality of the OCT system in detecting tumor boundaries between gliomas and brain tissue, gliomas and the optic nerve, schwannomas and peripheral nerves, and meningiomas and peripheral nerves.

For infiltrative tumor models, the OCT system can detect and distinguish glioma tissue from peritumoral normal brain tissue and the optic nerve. It provides an early warning of peritumoral normal brain tissue and optic nerve located deep beneath the tumor (within 2–3 mm). Additionally, the system allows for the detection of residual tumor in the tumor cavity after resection, thereby minimizing the residual tumor volume. A particularly significant aspect of this research is the application of the OCT system for detecting tumor boundaries in brainstem gliomas and its use in detecting boundaries between gliomas and peritumoral normal basal ganglia tissue when supratentorial gliomas invade the basal ganglia. There are three key reasons for the importance of this research: low feasibility and high difficulty of conducting similar studies in humans: Even if OCT‐based intraoperative detection of brainstem gliomas and gliomas invading the basal ganglia could generate OCT grayscale data, it is nearly impossible to obtain tissue specimens from these critical functional regions for pathological confirmation of the detected areas as brainstem or basal ganglia. Clinical significance of detecting glioma boundaries in these regions: The surgical treatment of intracranial tumors requires precision, and this is even more critical when addressing brainstem gliomas and gliomas invading the basal ganglia due to the need for protecting essential neural functions in these regions. Our study demonstrates that the OCT system can assist in identifying tumor boundaries during surgery in tumor‐bearing animal models of brainstem gliomas and gliomas invading the basal ganglia. This confirms the system's ability to support precision surgery for gliomas in critical functional regions. Lack of relevant research in the literature: To date, no studies have been reported in the domestic or international literature on the application of the OCT system for detecting tumor boundaries in brainstem gliomas or gliomas involving the basal ganglia in tumor‐bearing animal models [19]. This may be due to the deep location of these tumors and the high difficulty of surgically removing them, as well as the challenges of conducting in vivo OCT detection in these areas. This highlights the significance and value of our systematic research in applying the OCT system to detect tumor boundaries in various tumor models. Intraoperatively, brainstem fiber tracts are invisible to the surgeon, yet their integrity must be preserved to safeguard motor, sensory and other critical neurological functions. In the future, integrating OCT with tractography‐based identification and preservation of neural fiber bundles will represent a highly valuable avenue for both clinical practice and translational research.

For adhesive tumor models, the OCT system can detect and distinguish between gliomas and the optic nerve, schwannomas and the sciatic nerves, and meningiomas and the sciatic nerve. It provides an early warning for the optic and sciatic nerves located deep beneath the tumor (within 2–3 mm), reminding the surgeon to operate gently and minimize traction on the nerves, thereby maximizing the preservation of nerve function. We believe that the OCT system has a special role in adhesive tumor models. A review of the literature indicates that the application of the OCT system in intracranial adhesive tumor models has not yet attracted significant attention, research, or reporting. Our findings, particularly the early warning function of the OCT system for deep peripheral nerves in adhesive tumor models, demonstrate that the system not only plays a role in detecting tumor boundaries in infiltrative tumor models but also has the potential to identify tumor boundaries in adhesive tumor models during surgery. Unlike neurophysiological monitoring, which relies on direct stimulation of exposed nerves to determine their course during surgery, the accuracy of neurophysiological detection is significantly reduced for cranial nerves that are deeply embedded within or encased by the tumor tissue. This study confirms that the OCT system can alert the surgeon to the presence and course of cranial nerves while they are still located deep beneath the tumor. This suggests that the OCT system has the potential to become an alternative intraoperative technology for real‐time monitoring of cranial nerve pathways in tumor regions, similar to but with the added advantage of deep nerve warning capabilities. Additionally, this underscores the need to further enhance the performance of the OCT system, particularly in terms of increasing detection depth, to improve its ability to provide early warnings for adhesive tumor boundaries and to enhance its clinical value.

There are several limitations to this study. First, the OCT system was only tested on ex vivo human specimens, and the sample size was relatively small. Second, the application of machine learning techniques was not sufficiently in‐depth. In the future, we plan to expand the number of OCT‐detected specimens and enlarge the OCT grayscale value database for human intracranial tumors. We will also integrate machine learning techniques to automatically organize, analyze, and more rapidly identify tumor and nontumor tissues. Furthermore, we aim to initiate clinical trials as soon as possible to apply this technology to enable real‐time margin detection in live human intracranial tumors.

## 4 Conclusion

This study developed a hardware system based on handheld OCT technology for real‐time detection of neural system tumors and their boundaries. Additionally, a software system based on Visual C++ 6.0 was developed for the quantitative display of OCT grayscale images, enabling the visualization and reconstruction of grayscale images. A systematic database of OCT values were established based on various fresh ex vivo neural system tumor tissues and peritumoral nontumoral brain tissues were collected and measured using the OCT system. Statistically significant differences between the two tissue types were observed in OCT gray‐scale images with respect to light‐attenuation values, steepness of the A‐line trend, and the proportions of pixels within specific threshold ranges determined by a grid‐based method. Finally, applied to multiple in vivo tumor‐bearing animal models, our OCT system demonstrated its potential as an intraoperative tool for delineating neural tumor boundaries across diverse tumor types.

## 5 Materials and Methods

### 5.1 Specimen Collection

All specimens were freshly obtained from neurosurgical resections performed at West China Hospital, Sichuan University. After excision, the specimens were immediately placed in a 0°C portable refrigerated box and transported to the OCT detection system. The OCT scans were performed within 1 h of collection. The histological diagnosis of each specimen was confirmed through pathological examinations, including hematoxylin and eosin (HE) staining. Each specimen was labeled with a unique identifier, and the basic patient information—such as age, gender, laterality, lesion location, and final pathological diagnosis—was recorded. Peritumoral nontumoral tissue was defined as tissue at the tumor boundary, as identified by the surgeon through microscopic observation. This definition was further confirmed postoperatively by HE staining, ensuring the absence of tumor cells. The nature of the tumors was determined by pathologists, and the OCT images were compared with paraffin‐embedded sections from the same sample plane to assess whether the OCT images accurately reflected the tissue characteristics.

### 5.2 OCT Parameter Measurement

Each specimen was aligned with a reference marking board, and the system automatically identified the target region. The specimen was scanned in a 5.0 × 5.0 mm area at intervals of 0.1 mm, with the MEMS system performing ten back‐and‐forth scans. The OCT grayscale values of the tumor region and peritumoral nontumoral brain tissue were measured separately. Grayscale values were set with a maximum brightness of 255 and a minimum of 0.

(A) *Light attenuation coefficient analysis*. The light attenuation coefficient of the specimen was calculated, and statistical methods were used to compare the light attenuation coefficients of tumor regions and peritumoral nontumoral brain tissues across multiple specimens. The appropriate threshold for the light attenuation coefficient was determined to ensure high sensitivity and specificity of the OCT system in distinguishing between brain tumor tissue and peritumoral nontumoral brain tissue.

The formula for calculating the light attenuation coefficient (*μ*_t_) is as follows:

$$\mu t=\frac{10}{L}\log\left(\frac{p_{i}}{P_{L}}\right)\left(dB/mm\right)$$

where log refers to the base‐10 logarithm. *P_i_* ​ represents the grayscale value at the starting row (where the grayscale value is high), and *P_L_* ​ represents the grayscale value at the ending row (where the grayscale value is between 40 and 80). *L* is the distance between the starting and ending rows, calculated as:

L=numberofrows×0.024445mm

If the calculated value exceeds 11, it indicates that the starting row may be a strong reflection noise point or the ending row may be a low grayscale noise point, and this value is discarded. If the calculated value is negative, it indicates that the ending row may be a high grayscale noise point, and this value is also discarded.

(B) *A‐line and trendline analysis*. The OCT B‐scan grayscale image corresponds to a specific matrix of grayscale values. A single column of pixel grayscale values is selected, and these values are assigned to the vertical axis, with the horizontal axis representing the depth of the specimen, forming an A‐line. The A‐line can be used to determine the distance between the OCT probe and the specimen, as well as the grayscale peak values. To smooth the data, the grayscale values along the A‐line are averaged every 10 rows, forming a smooth curve known as the trendline. The trendline is used to obtain the peak value and the slope of the descending segment. The aspect ratio of the trendline is adjusted to 4:3, and ImageJ software is used to measure the angle between the descending segment of the trendline and the vertical axis.

(C) *Analysis of total pixel count within specific thresholds using grid method*. Using ImageJ software, the grayscale image to be analyzed is opened, and a section along the midline of the top layer of the specimen's grayscale image is selected within a 55 × 100 resolution area. The image type is set to 8‐bit format, and the threshold is adjusted. The threshold range is adjusted to 70–140 and 70–110, respectively. ImageJ software is then used to calculate the total number of pixels within the selected threshold range.

### 5.3 Development of Intraoperative OCT Visualization System for Neural Tumors and their Boundaries

Further, we conducted grayscale image visualization studies based on OCT scanning and performed 3D visual reconstruction of the grayscale images from the actual OCT scanning regions.

After scanning with the OCT system, the raw output is a grayscale image. Each OCT B‐scan grayscale image was pseudo‐colored in ImageJ using a 16‐color lookup table to generate calibrated, color‐coded maps. Each gray‐value was mapped to a distinct hue—red, orange, yellow, green, blue, and black—producing a 2D, false‐color B‐scan. In this color scale, the transition from red (high gray‐value) to black (low gray‐value) corresponds to high‐ to low‐signal intensities. Since the grayscale range is 0–255, we treated 255 as the maximum intensity.

Each 5 × 5 × 4 mm specimen generated 200–500 raw B‐scan grayscale images. These images were imported into MATLAB for data recognition, processing, and analysis. A MEMS mirror scanned the tissue in a bidirectional raster pattern with a step size of 0.1 mm; consecutive B‐scans were therefore separated by 0.1 mm. Owing to the high axial resolution of OCT, even when the probe returned to the same lateral position, the resulting images exhibited minute differences. We exploited these differences to enhance spatial resolution by an “interleaving” strategy. Grayscale images acquired during five bidirectional passes were interleaved to fill the 0.1 mm gaps left by unidirectional scanning. Nine additional repeated scans at each location were then sequentially inserted into the corresponding gaps, yielding a final voxel size of 0.1 × 0.1 × 0.1 mm.

For every B‐scan, each A‐line (column) was processed:

- Extract the column → sort pixel values → select the fourth largest value → compute the running mean of every five consecutive pixels → if the mean is <45, calculate the attenuation coefficient; otherwise slide one pixel and repeat.

The resulting attenuation coefficients of all images were assembled into a 3D matrix.

Binarize each image (threshold 70) → apply morphological opening/closing → locate the first “1” in each column to obtain the tissue surface index → compile indices into a surface matrix. Four lateral faces were created; upper voxels outside the surface index were set to NaN to ensure seamless joining with the top surface. Based on the attenuation matrix, voxels were colored in three zones:

- Red (0–5): darker red near 0, brighter red near 5.
- Yellow (5–6): transition zone.
- Green (6–11): darker green near 6, brighter green near 11.

Values outside 0–11 or NaN were rendered white and excluded.

Side faces were binarized (>70), then colored with the same palette according to the attenuation value at each surface voxel. The final 3D volume depicts tumor (red), nontumor (green), and transition (yellow) regions with isotropic 0.1 mm resolution.

Comprehensive experimental protocols for the construction of the swept‐source OCT system, software development, and animal model studies are provided in the Supporting Information.

## Author Contributions

Jiuhong Li, Jinwei Li, and Gonggong Lu contributed equally to this work as cofirst authors. They were primarily responsible for the experimental design, implementation, and data analysis. Feilong Yang and Jing Li supported the development and optimization of the OCT system and performed hardware testing. Xin Qi and Rui Zhang provided technical expertise in animal model construction and intraoperative imaging. Xiang Li and Jiachen Sun contributed to data acquisition and statistical analysis, as well as manuscript drafting. Haibo Rao provided critical insights into OCT system engineering and imaging algorithms. Gonggong Lu assisted with the pathological validation of the findings. Xuhui Hui and Si Zhang supervised the project, drafted and reviewed the manuscript, and provided funding and resources to ensure the successful completion of the study. All authors read and approved the final manuscript.

## Ethics Statement

Informed consent was obtained from all participants in the study. The collection and study design for all patient specimens received approval from the Biomedical Ethics Committee of West China Hospital, Sichuan University (Approval No.: 2024. 2180). All animal experiments included in our study were approved by Institutional Animal Care and Use Committee of West China Hospital of Sichuan University (Approval No.: 20220505004).

## Funding Information

This work was supported by the Innovation Research Program of Sichuan University (No. 2022SCUH0019), Program of Science & Technology Department of Sichuan Province (No. 2018SZ0043), the Postdoctoral Research and Development Fund of West China Hospital, Sichuan University (No. 2023HXBH052), Promotion Association for Sichuan Science and Education (No. KJXC24‐0319), and Health Commission of Mianyang (No. 202347 and No. 2024032).

## Conflicts of Interest

Author Jing Li is an employee in Chengdu Incrpeak Optoelectronics Technology Co., Ltd, but has no potential relevant financial or nonfinancial interests to disclose. The other authors declare no conflicts of interest.

## Supporting information



Supporting Information

## Data Availability

The datasets generated and/or analyzed during the current study are available from the corresponding authors, H. B. Rao., X. H. Hui., and S. Zhang., upon reasonable request.
