# Supplementary material for: Preliminary Establishment of a Method and System for Detecting Neural Tumor Boundaries Based on Optical Coherence Tomography
Source: MedComm (2020). 2025 Nov 28;6(12):e70498. doi: 10.1002/mco2.70498 (PMC12662810; doi:10.1002/mco2.70498)
Supplement: Supplementary file 1 — Supporting Information [file MCO2-6-e70498-s001.docx]

**Preliminary Establishment of a Method and System for Detecting Neural Tumor Boundaries Based on Optical Coherence Tomography**

Jiuhong Li^1#^, Jinwei Li^1#^, Gonggong Lu^1#^, Feilong Yang^1, 2^, Jing Li^3^, Xin Qi^4^, Rui Zhang^1^, Xiang Li^1^, Jiachen Sun^1^, Haibo Rao^5^*, Xuhui Hui^1^*, Si Zhang^1^*.

^1^Department of Neurosurgery, Department of Cardiovascular Surgery, West China Hospital of Sichuan University, Chengdu, 610041, China.

^2^Department of neurosurgery, the Affiliated Santai Hospital of North Sichuan Medical College, Mianyang, 621100, China.

^3^Chengdu Incrpeak Optoelectronics Technology Co., Ltd. Central bldg., Optoelectric Industrial Park, No. 187, Sec. 1, Changcheng Rd., Chengdu, 610207, China.

^4^Laboratory of Neurosurgical Institute, West China West China Hospital of Sichuan University, Chengdu, 610041, China.

^5^School of Optoelectronic Science and Engineering of UESTC, University of Electronic Science and Technology of China, Chengdu, 611731, China.

**^#^These authors contributed equally to this work as co-first authors.**

***Correspondence:**

Haibo Rao rhb@uestc.edu.cn

School of Optoelectronic Science and Engineering of UESTC, University of Electronic Science and Technology of China, Chengdu, 611731, China.

Xuhui Hui huixuhuiwc@126.com

Department of Neurosurgery, Department of Cardiovascular Surgery, West China Hospital of Sichuan University, Chengdu, 610041, China.

Si Zhang zhangsi198712@126.com

Department of Neurosurgery, Department of Cardiovascular Surgery, West China Hospital of Sichuan University, Chengdu, 610041, China.

**Table S1. Basic information of human fresh *ex vivo* brain tissues involving the tumor boundary for OCT detection.**

| Patient No. | Location | Noncancerous OCT volumes | Cancer-infiltrated OCT volumes | Tumor type | Grade of cancer | Diagnosis | Age (year) | Gender |
| --- | --- | --- | --- | --- | --- | --- | --- | --- |
| Patient-1 | 1A | - | 10 | Glioma | Ⅱ | Left frontal diffuse astrocytoma | 26 | Male |
|  | 1B | 2 | - | - | - | Left frontal diffuse astrocytoma | 26 | Male |
| Patient-2 | 2A | - | 5 | Glioma | Ⅱ | Left frontal diffuse astrocytoma | 75 | Male |
|  | 2B | 3 | - | - | - | Left frontal diffuse astrocytoma | 75 | Male |
| Patient-3 | 3A | - | 2 | Glioma | Ⅱ | Oligodendroglioma located at bilateral frontal lobe corpus callosum | 46 | Male |
| Patient-4 | 4A | - | 4 | Glioma | Ⅱ | Right central low-grade glioma | 43 | Male |
| Patient-5 | 5A | - | 5 | Glioma | Ⅲ | Left occipital anaplastic oligodendroglioma | 62 | Male |
|  | 5B | 2 | - | - | - | Left occipital anaplastic oligodendroglioma | 62 | Male |
| Patient-6 | 6A | - | 8 | Glioma | Ⅲ | Right frontal anaplastic astrocytoma | 45 | Female |
|  | 6B | 1 | - | - | - | Right frontal anaplastic astrocytoma | 45 | Female |
| Patient-7 | 7A | - | 6 | Glioma | Ⅲ | Right frontal recurrent anaplastic oligodendroglioma | 46 | Female |
| Patient-8 | 8A | - | 4 | Glioma | Ⅳ | Left frontal recurrent glioblastoma | 36 | Female |
| Patient-9 | 9A | - | 7 | Glioma | Ⅳ | Left temporal and insular glioblastoma | 34 | Female |
|  | 9B | 6 | - | - | - | Left temporal and insular glioblastoma | 34 | Female |
| Patient-10 | 10A | - | 1 | Glioma | Ⅳ | Right frontal glioblastoma | 70 | Female |
|  | 10B | 5 | - | - | - | Right frontal glioblastoma | 70 | Female |
| Patient-11 | 11A | - | 1 | Glioma | Ⅳ | Right temporal glioblastoma | 38 | Male |
| Patient-12 | 12A | - | 1 | Glioma | Ⅳ | Diffuse midline glioma located at the left basal ganglia | 11 | Male |
| Patient-13 | 13A | - | 6 | Glioma | Ⅳ | Right frontotemporal insular glioblastoma | 53 | Female |
|  | 13B | 2 | - | - | - | Right frontotemporal insular glioblastoma | 53 | Female |
| Patient-14 | 14A | - | 1 | Glioma | Ⅱ | Right cerebellar vermis diffuse astrocytoma | 3 | Male |
| Patient-15 | 15A | 1* | - | - | - | Right vestibular schwannoma | 50 | Female |
| Patient-16 | 16A | 1* | - | - | - | Left cerebellopontine angle hemangioblastoma | 59 | Female |
| Patient-17 | 17A | 1* | - | - | - | Left cerebellopontine angle meningioma | 48 | Male |
| Patient-18 | 18A | - | 1 | Metastasis | - | Left temporal hippocampus metastatic lung cancer | 66 | Female |
|  | 18B | 2 | - | - | - | Left temporal hippocampus metastatic lung cancer | 66 | Female |
| Patient-19 | 19A | - | 9 | Metastasis | - | Right cerebellar metastatic lung cancer | 65 | Male |
|  | 19B | 3* | - | - | - | Right cerebellar metastatic lung cancer | 65 | Male |
| Patient-20 | 20A | - | 1 | Lymphoma | - | Right parietal diffuse large B-cell lymphoma | 63 | Male |
|  | 20B | 2 | - | - | - | Right parietal diffuse large B-cell lymphoma | 63 | Male |

No. number; *cerebellum. OCT, optical coherence tomography.

**Table S2. ROC curve results for light attenuation values of supratentorial gliomas and peri-tumoral non-tumoral brain tissue.**

| Group | Cut-off point | Sensitivity | Specificity | AUC |
| --- | --- | --- | --- | --- |
| WHO grade 4 gliomas and peri-tumoral non-tumoral brain tissue | 5.75 mm^-1^ | 0.900 | 0.923 | 0.956 |
| WHO grade 3 gliomas and peri-tumoral non-tumoral brain tissue | 5.95 mm^-1^ | 0.737 | 1.000 | 0.860 |
| WHO grade 2 gliomas and peri-tumoral non-tumoral brain tissue | 5.95 mm^-1^ | 0.429 | 1.000 | 0.657 |
| High-grade gliomas and peri-tumoral non-tumoral brain tissue | 5.57 mm^-1^ | 0.795 | 0.937 | 0.934 |
| High- and low-grade gliomas and peri-tumoral non-tumoral brain tissue | 5.57 mm^-1^ | 0.650 | 0.952 | 0.846 |

High-grade gliomas refer to WHO grade 3 and 4 gliomas, while high- and low-grade gliomas include WHO grade 2, 3, and 4 gliomas. AUC, area under curve; ROC, receiver operating characteristic; WHO, World Health Organization.

**Table S3. ROC curve results for A-line and its trend line at different working distances between gliomas and peri-tumoral non-tumoral brain tissue.**

| Group | Cut-off point | Sensitivity | Specificity | AUC |
| --- | --- | --- | --- | --- |
| Any distance between the probe and the specimen surface | 9.62° | 0.947 | 0.5 | 0.762 |
| The distance between the probe and the specimen surface is ≤1.5 mm | 16.1° | 0.526 | 0.923 | 0.777 |
| The distance between the probe and the specimen surface is >1.5 mm | 9.62° | 0.921 | 0.579 | 0.751 |

ROC, receiver operating characteristic.

**Table S4. ROC curve results for the 70–140 gray-value range within 55 × 100 pixel regions in gliomas and surrounding non-tumoral brain tissue.**

| Group | Cut-off point | Sensitivity | specificity | 70-140 Area under the gray range curve |
| --- | --- | --- | --- | --- |
| WHO grade 4 gliomas and peri-tumoral non-tumoral brain tissue | 1184 | 0.75 | 0.789 | 0.771 |
| WHO grade 3 Gliomas and peri-tumoral non-tumoral brain tissue | 1073.5 | 0.947 | 0.632 | 0.809 |
| WHO grade 2 gliomas and peri-tumoral non-tumoral brain tissue | 1065.5 | 0.897 | 0.632 | 0.789 |
| High-grade gliomas and peri-tumoral non-tumoral brain tissue | 1184 | 0.567 | 0.789 | 0.698 |

High-grade gliomas refer to WHO grade 3 and 4 gliomas, while high- and low-grade gliomas include WHO grade 2, 3, and 4 gliomas; AUC, area under curve; ROC, receiver operating characteristic; WHO, World Health Organization.

**Table S5. Pixel counts using the grid method of *in vivo* OCT detection for animal tumors and peri-tumoral normal tissue.**

| Group | *n* | Pixel count for grayscale values 100–140 | *p* value |
| --- | --- | --- | --- |
| Supratentorial C6 Tumor | | | |
| Tumor region | 15 | 92.47 ± 198.25 | 0.002 |
| Peri-tumoral normal brain tissue | 15 | 472.33 ± 372.51 | - |
| Optic Chiasm C6 Tumor | | | |
| Tumor region | 12 | 87.58 ± 101.27 | 0.005 |
| Optic nerve | 12 | 401.25 ± 309.54 | - |
| Brainstem C6 Tumor | | | |
| Tumor region | 16 | 101.37 ± 93.55 | <0.001 |
| Peri-tumoral normal brainstem tissue | 16 | 393.69 ± 179.73 | - |
| Basal ganglia C6 tumor | | | |
| Tumor region | 6 | 141.50 ± 60.07 | 0.001 |
| Peri-tumoral normal basal ganglia tissue | 6 | 482.33 ± 162.60 | - |
| Cerebellar C6 tumor | | | |
| Tumor region | 8 | 144.75 ± 214.30 | 0.064 |
| Peri-tumoral normal brain tissue | 8 | 435.38 ± 347.76 | - |
| Supratentorial U87 tumor | | | |
| Tumor region | 14 | 64.36 ± 60.44 | <0.001 |
| Peri-tumoral normal brain tissue | 14 | 512.29 ± 273.04 | - |

OCT, optical coherence tomography.

**Table S6. Mean grayscale value of *in vivo* OCT detection for animal tumors and peri-tumoral normal tissue.**

| Group | *n* | Mean grayscale value | *p* value |
| --- | --- | --- | --- |
| Supratentorial C6 Tumor | | | |
| Tumor region | 13 | 58.73 ± 16.67 | 0.014 |
| Peri-tumoral normal brain tissue | 13 | 74.34 ± 12.95 | - |
| Optic chiasm C6 Tumor | | | |
| Tumor region | 12 | 45.30 ± 14.09 | 0.027 |
| Optic nerve | 12 | 59.23 ± 14.60 | - |
| Brainstem C6 Tumor | | | |
| Tumor region | 17 | 59.46 ± 11.34 | 0.010 |
| Peri-tumoral normal brainstem tissue | 17 | 71.11 ± 13.24 | - |
| Basal ganglia C6 tumor |  |  |  |
| Tumor region | 7 | 64.37 ± 13.26 | 0.073 |
| Peri-tumoral normal basal ganglia tissue | 7 | 79.16 ± 14.83 | - |
| Cerebellar C6 tumor | | | |
| Tumor region | 7 | 57.94 ± 8.56 | 0.512 |
| Peri-tumoral normal brain tissue | 7 | 64.24 ± 22.69 | - |
| Supratentorial U87 tumor | | | |
| Tumor region | 15 | 61.31 ± 16.47 | 0.007 |
| Peri-tumoral normal brain tissue | 16 | 76.71 ± 13.17 | - |

OCT, optical coherence tomography.

**Supplementary Materials and Methods**

**2.1 Creation of the Intraoperative Swept-Source OCT System for Brain Tumors**

An intraoperative swept-source optical coherence tomography (OCT) system was developed based on the principles of low-coherence interferometry and Fourier transform, a theoretical model for the swept-source optical coherence tomography (OCT) (HSL-20) system was established^1^. The system operates with a central wavelength of 1310 nm and uses a MEMS-based handheld probe optimized for portability and ease of use in the surgical environment. The effective scanning area of the system includes a depth of 11 mm and a width of 5 mm, allowing precise detection of both tumor tissue and its boundaries. The overall system design, including the functional block diagram, hardware specifications, and imaging software, is described in detail in the Supplementary Materials and Methods.

The system was constructed to ensure continuous, high-resolution imaging during surgery, enabling the surgeon to monitor tumor boundaries in real-time. The OCT system was then validated using a series of *ex vivo* and *in vivo* experiments, focusing on its capability to differentiate between tumor and non-tumoral brain tissues based on light attenuation values and grayscale analysis.

**2.2 Intraoperative Swept-Source OCT Imaging Software Design**

The software for the swept-source OCT system includes both the development of the hardware's accompanying software and the establishment of the optical parameter model and algorithm for brain glioma tissues. The system software was designed in a modular format, comprising several key components: timing control, data acquisition, data processing, and image reconstruction. Emphasis was placed on ensuring the software's usability, reliability, and stability. A physical model and algorithm were developed to rapidly and accurately acquire optical parameters from highly scattering biological tissues. Additionally, diagnostic threshold standards with high sensitivity and specificity, meeting pathological requirements, were established to optimize the accuracy of tumor boundary identification.

By extracting the interference pattern from the spectral linewidth signal (intensity absorption) and converting it through software algorithms, tissue stratification information is reconstructed and displayed on the computer. Tumor tissues and peri-tumoral non-tumoral brain tissues exhibit different reflection, absorption, and transmission characteristics of the spectral intensity signals. These differences result in distinct grayscale values and variation patterns, which are used to differentiate between tissue types.

**2.3** **Establishment of Animal Models of Neural Tumors**

To perform *in vivo* OCT imaging scans, we constructed several tumor-bearing animal models, including rat brain glioma models, optic chiasm glioma models, sciatic nerve schwannoma models, as well as nude mouse brain glioma and sciatic nerve meningioma models. The specific modeling methods are described in the Supplementary Materials. All animal surgeries were approved by the Animal Protection and Utilization Committee of Sichuan University.

The C6 rat glioma cell line was obtained from the Shanghai Cell Bank, part of the Typical Culture Preservation Committee of the Chinese Academy of Sciences. The U87MG human astrocytoma cell line and the IOMM-Lee human malignant meningioma cell line are preserved at the Chengdu Tianfu Life Science Park. The RT4-D6P2T (referred to as RT4) rat malignant schwannoma cell line was acquired from the American Type Culture Collection (ATCC).

Each cell line is utilized for specific model establishment:

- **C6 Cell Line**: Used to develop models of brain glioma and optic pathway glioma in SD rats.
- **U87MG Cell Line**: Employed to establish a supratentorial glioma model in BALB/C nude mice.
- **IOMM-Lee Cell Line**: Utilized for creating models of meningioma and sciatic nerve adhesion in BALB/C nude mice.
- **RT4 Cell Line**: Applied in developing models of sciatic nerve schwannoma in SD rats.

The experimental animals used include: SD Rats: All female, 10 weeks old, weighing between 210-220 g, purchased from Chengdu Ensiweier Biotechnology Co., Ltd. BALB/C Nude Mice: All female, 5 weeks old, weighing between 20-25 g, also obtained from Chengdu Ensiweier Biotechnology Co., Ltd. Both types of animals were housed in separate cages under controlled temperature and humidity conditions, with free access to food and water, and a 12-hour light/dark cycle.

The commercially obtained C6 cells were sourced from the Shanghai Cell Bank of the Typical Culture Preservation Committee, Chinese Academy of Sciences. After being revived by the supplier, the cells were adhered to culture flasks and shipped aseptically with medium. Upon arrival, the cells were cultured in a 37°C, 5% CO₂ incubator for 24 hours. The medium was refreshed every 2-3 days based on cell growth and medium color changes. When the cells reached 80-90% confluency as observed under a microscope, they were subcultured at a ratio of 1:2 to 1:3. Subculture Procedure: Preheat 0.25% trypsin-0.53 mM EDTA solution in a 37°C water bath. Remove the medium from the flask, add 4 mL of PBS to the flask, gently shake, and discard the PBS. Add 2 mL of preheated trypsin to the flask and incubate at 37°C for 1 minute. Terminate the digestion by adding 2 mL of complete medium. Use a pipette to gently detach cells from the flask wall and collect the cell suspension. Centrifuge at 800 rpm for 3 minutes, discard the supernatant, resuspend the cells in complete medium, and transfer them to a 10 cm culture dish for further subculturing. The subcultured C6 cells were maintained in high-glucose DMEM supplemented with 10% fetal bovine serum (Umedium He Fei China) in a 37°C, 5% CO₂ incubator. The medium was replaced every 1-2 days depending on growth, with daily monitoring and recording of cell conditions, including state, density, and morphology. Once cell growth was stable, the cells were prepared for use in modeling gliomas and optic chiasm gliomas in SD rats.

In addition to the standard steps in cell culture, such as cell rinsing, EDTA digestion, and centrifugation, the cell preparation process for tumor modeling also includes additional steps like PBS washing, cell counting, and cell concentration adjustment post-centrifugation. Residual trypsin, cell debris, and DMEM in the tumor cells following centrifugation are removed by repeated PBS washing. This additional washing step helps reduce localized immune or inflammatory responses after cell implantation.

To prepare the supratentorial parenchymal glioma model in SD rats, C6 cells in the logarithmic growth phase were digested and resuspended in 5 mL of PBS. A 10 μL sample was taken for cell counting, and based on the results, the concentration of viable cells was adjusted to 1×10⁴ cells/μL. The injection site was determined using the bregma as the origin (X=0, Y=0), with coordinates set to X=-3.50 mm (negative indicating the right side) and Y=1.00 mm on the skull. After drilling through the skull at this location with a RWD microdrill, a 10 μL microsyringe was positioned with its needle tip against the dura mater at the drill site. The Z-axis was zeroed at this point, and the needle was advanced into the brain until Z=6.00 mm, then retracted by 1.00 mm before injecting the tumor cell suspension. The injection volume was 10 μL at a rate of 1 μL/min. After injection, the needle was left in place for 10 minutes before being slowly withdrawn. Bone wax was applied to seal the hole, the scalp was sutured, and the area was disinfected, with animals maintained under standard conditions.

For the optic chiasm glioma model in SD rats, the concentration of viable cells was adjusted to 5×10⁴ cells/μL based on the cell count. The injection site was also based on the bregma (X=0, Y=0), with drilling performed at this location. The syringe needle was positioned at the dura mater after drilling, zeroed on the Z-axis, and advanced into the brain to Z=9.50 mm, where injection was initiated. The injection volume was set to 2 μL at a rate of 0.5 μL/min. Following injection, the needle was left in place for 4 minutes before being gradually withdrawn.

For the brainstem glioma model in SD rats, the concentration of viable cells was adjusted to 5×10⁴ cells/μL. Using the bregma as the origin (X=0, Y=0), the injection site was set to X=-2.00 mm, Y=-10.56 mm (negative indicating the caudal direction), and Z=8.50 mm. The injection volume was 2 μL, with an injection speed of 0.5 μL/min. After injection, the needle was left in place for 4 minutes before being slowly withdrawn. For the cerebellar glioma model in SD rats, the concentration of viable cells was adjusted to 1×10⁴ cells/μL. Using the bregma as the origin, the injection site was set to X=-2.00 mm, Y=-11.04 mm, and Z=4.00 mm. The injection volume was 10 μL, with an injection speed of 1 μL/min. After injection, the needle was left in place for 10 minutes before being slowly withdrawn.

The commercially obtained RT4 cells were purchased from the American Type Culture Collection (ATCC). Following revival by the supplier, the cells were adhered to culture flasks, filled with medium, and shipped aseptically. The subsequent cell culture procedures were similar to those for C6 cells, with specific adjustments to account for the unique characteristics of RT4 cells. Due to their larger size and cytoplasm-rich nature, RT4 cells require greater centrifugal force and longer centrifugation time, with parameters set to 1000 rpm for 5 minutes. Residual trypsin, cell debris, and DMEM were removed by multiple PBS washes after centrifugation. Additionally, because RT4 cells have more elongated and complex dendritic structures and adhere more tightly to the culture dish, the digestion time was extended to 3 minutes. Once the cells exhibited stable growth conditions, they were prepared for modeling sciatic nerve schwannoma in SD rats.

RT4 cells in the logarithmic growth phase were digested and resuspended in 5 mL of PBS. A 10 μL sample was taken for cell counting, and the viable cell concentration was adjusted to 1×10⁴ cells/μL. Following anesthesia induction and maintenance using a face mask, the SD rats were fixed on a temperature-controlled stage. After preparing the surgical site aseptically, a skin incision was made along the premarked lines, and the skin and muscle layers were opened step by step. The sciatic nerve was exposed through the intermuscular space, freed, and stabilized using microforceps or a dissector. Under a microscope, the RT4 cell suspension was slowly injected into the sciatic nerve. The needle was withdrawn slowly 1 minute after injection.

After revival, the cells were adhered to culture dishes and cultured similarly to the C6 cell line. Once stable growth conditions were achieved, the cells were prepared for modeling gliomas in nude mice. The concentration of viable U87MG cells was adjusted to 1×10⁴ cells/μL, following the same preparation steps as for C6 cells. The specific procedural steps are outlined in Figure 2-16. The stereotaxic frame on the RWD apparatus was modified to include a mouse-specific adapter to secure the nude mouse and maintain anesthesia. The injection site was set to coordinates X=-1.00 mm, Y=-1.34 mm, and Z=3.00 mm. A total of 10 μL of cell suspension was injected. All other procedures were consistent with the stereotaxic intracranial tumor implantation process used for SD rats.

After revival, the cells adhered to culture dishes and were cultured following protocols similar to those for the C6 cell line. Once cell growth stabilized, the cells were prepared for modeling sciatic nerve meningiomas in nude mice. The viable cell concentration was adjusted to 1×10⁴ cells/μL, using the same preparation steps as for C6 cells. The stereotaxic frame on the RWD apparatus was adapted with a mouse-specific adapter to secure the nude mouse and maintain anesthesia. A total of 10 μL of the cell suspension was injected, following the same protocol as for RT4 tumor cell implantation in the sciatic nerve of SD rats.

**2.4** **Intraoperative Tumor Boundary Detection in Live Animal Models using the OCT System**

Two to three weeks after tumor implantation, the OCT system was used to detect tumor boundaries in the live animal models, validating the system’s effectiveness and accuracy. The detailed procedures and materials are described in the Supplementary Methods and Materials (Supplement Method). When the residual tumor thickness was less than 3 mm, the OCT system could detect the normal brain tissue or cranial nerves located beneath the tumor, providing an early warning regarding the deep regions of the tumor and the normal brain tissue or cranial nerves in those areas. At this point, the surgeon continued tumor reduction under the microscope, removing approximately 2 mm of tumor tissue and progressively reducing the residual tumor thickness to less than 1 mm. Once the residual tumor was resected, the OCT system was used for real-time detection of the tumor cavity during surgery. If the OCT imaging revealed that the residual tumor thickness in a specific area exceeded 1 mm, the surgeon carefully resected the tumor further to minimize the residual thickness to below 1 mm. In summary, intraoperative real-time OCT monitoring was used to guide tumor reduction, keeping the residual tumor thickness within the tumor cavity to less than 1 mm in most regions. When the OCT system showed that the residual tumor thickness in the majority of the tumor cavity was controlled within 1 mm, the specimen from the detected area was collected and fixed. For tumor-bearing animal models involving sciatic nerve infiltration and adhesion, a similar surgical procedure was followed.

During the OCT system's detection and tumor resection process, paraffin sections were prepared from the following tissues: supratentorial gliomas and peri-tumoral normal brain tissue, gliomas and peri-tumoral normal cerebellar tissue, gliomas and peri-tumoral normal brainstem tissue, gliomas and peri-tumoral normal basal ganglia tissue, sciatic nerve schwannomas from SD rats. Additionally, paraffin sections were prepared from human gliomas and peri-tumoral normal brain tissue, as well as sciatic nerve meningiomas from BALB/c nude mice. All sections were subjected to hematoxylin and eosin (HE) staining for histopathological analysis.

**2.5 Data Analysis Software**

All data in this study are presented as mean ± standard deviation (SD). Comparisons of means between two samples were conducted using the t-test, with statistical significance set at *p* < 0.05. All statistical analyses and receiver operating characteristic (ROC) curve plotting were performed using SPSS software (Version 23.0; Microsoft Corp.).

**REFERENCES**

1. Aumann S, Donner S, Fischer J, Müller F. Optical Coherence Tomography (OCT): Principle and Technical Realization. In: Bille JF, ed. *High Resolution Imaging in Microscopy and Ophthalmology: New Frontiers in Biomedical Optics*. Springer

Copyright 2019, The Author(s). 2019:59-85.
